# Hysteroscopy for retained products of conception: a single-institution experience

**DOI:** 10.1186/s12905-023-02170-0

**Published:** 2023-01-18

**Authors:** Ling Han, Gang Shi, Ai Zheng, Jiaying Ruan

**Affiliations:** 1 Department of Obstetrics and Gynecology, West China Second Hospital, Sichuan University, Renmin South Road, Chengdu, Sichuan China; 2 Key Laboratory of Birth Defects and Related Diseases of Women and Children (Sichuan University), Ministry of Education, Chengdu, China

**Keywords:** Complete removal rate, Menstruation, Hysteroscopy, Retained products of conception

## Abstract

**Background:**

Retained products of conception can occur with induced abortion during early-term pregnancy, induction of labor during mid-term pregnancy, drug-induced abortion, miscarriage, cesarean delivery, or full-term normal delivery. Compared with traditional dilation and curettage, hysteroscopy is a safer and more effective treatment method for retained products of conception. This study aimed to report the efficacy of hysteroscopy for treating retained products of conception and to share our new clinical perspectives.

**Methods:**

This retrospective, single-center study was conducted at a tertiary hospital in Chengdu, China. We included 36 patients with retained products of conception who underwent hysteroscopy at our hospital.

**Results:**

Our study reported a complete removal rate of 80.5% (29/36) with one procedure. The normal menstruation recovery rate during 1 year of follow-up was 91.6% (33/36). A low rate of postoperative intrauterine adhesions (2.8% [1/36]) was also reported.

**Conclusion:**

Our retrospective study elucidated the use of hysteroscopy for retained products of conception. We also shared new perspectives regarding hysteroscopy and optimal surgical methods for treating retained products of conception as well as our experience treating residual products with hysteroscopy. To our knowledge, no other study has shared similar experiences.

## Background

Retained products of conception (RPOC) refer to placental or fetal tissue that can occur with induced abortion during early-term pregnancy, induction of labor during mid-term pregnancy, drug-induced abortion, miscarriage, cesarean delivery, or full-term normal delivery [1]. RPOC can result in normal placentation, the placenta accreta spectrum (PAS). PAS may be suspected antenatally through ultrasound or magnetic resonance imaging (MRI) or confirmed when placental delivery is difficult [2]. If PAS is not managed immediately after delivery, conservative management can be considered according to the clinical conditions. However, the treatment method used after ultrasound imaging includes dilation and curettage, which is a blind surgical procedure that is associated with complications such as intrauterine adhesion, incomplete removal, and uterine perforation [3]. On the contrary, hysteroscopy is a safer and more effective treatment method for RPOC [4]. The purpose of this study was to determine the efficacy of hysteroscopy for treating RPOC.

## Methods

This retrospective, single-center study was conducted at a tertiary hospital in Chengdu, China. A cohort of women diagnosed with RPOC between January 1, 2018 and December 1, 2021, were included in the study. The study was approved by the Ethics Committee of the West China Second University Hospital, Sichuan University.

All diagnoses were determined using ultrasound examination. Preoperative MRI was performed to exclude placenta accreta and arteriovenous fistula. Hysteroscopy included the cold knife and heat loop excision techniques. All patients were treated by one skilled physician to eliminate the influence of differences in skill. The objective of this study was to report the clinical efficacy and complications of hysteroscopy for treating RPOC.

All patients were followed up at 1 month after surgery. At this time, an ultrasound examination was performed to assess whether complete removal was achieved. All patients were followed up for 3 months to 1 year at the outpatient clinic to evaluate their menstruation condition and to determine whether postoperative intrauterine adhesion occurred.

## Results

Table 1 provides an overview of the patients’ characteristics and outcomes of hysteroscopy. We included 36 patients with RPOC who underwent hysteroscopy at our hospital. The mean age of the patients was 27 (range 24 to 40) years. Perioperative bleeding ranged from 5 to 1200 mL. The methods of pregnancy termination were as follows: induced abortion during early-term pregnancy, 15 patients; induced abortion during mid-term pregnancy, 7 patients; drug-induced abortion, 5 patients; cesarean delivery, 5 patients; and full-term normal delivery, 4 patients. Six patients required manual removal of the placenta. Two patients who underwent induced abortion during mid-term pregnancy required manual removal of the placenta. Of the patients with RPOC who gave birth via cesarean delivery, two required manual removal of the placenta during surgery because of placenta adhesion. Of the four patients who experienced full-term normal delivery, two required manual removal of the placenta because of placenta adhesion, one had partial placenta implantation, and one had a retained placenta. Before hysteroscopy, three patients had already undergone curettage to remove the intrauterine placenta. One patient with a cesarean scar pregnancy underwent curettage after uterine arterial embolization. One patient who underwent induced abortion during mid-term pregnancy also underwent curettage after manual removal of the placenta because of placenta adhesion. One patient underwent curettage because of a retained placenta after full-term normal delivery. The examination revealed negative human chorionic gonadotropin levels for 26 patients and positive human chorionic gonadotropin levels ranging from 17 to 7092.0 mIU/mL for 10 patients. All patients underwent color ultrasound and MRI examinations. Four patients were examined using uterine arteriography because of a suspected uterine arteriovenous fistula. According to the color ultrasound and MRI results, the largest residual product was 6.0 × 5.9 × 5.7 cm, and the smallest residual product was 1.0 × 0.7 × 1.3 cm.

**Table 1:** Overview of patient characteristics and outcomes of hysteroscopy

Patient characteristics and outcomes of hysteroscopy	Value
Patient characteristics	
Age, years	27 (range 24–40)
Methods of pregnancy termination	
Induced abortion during early-term pregnancy	15
Induced abortion during mid-term pregnancy	7
Drug-induced abortion	5
Cesarean delivery	5
Full-term normal delivery	4
Intervention before hysteroscopy	
Manual removal of the placenta	4
Curettage	3
Preoperative examination of hysteroscopy	
Color ultrasound and MRI	36
Color ultrasound, MRI, and uterine arteriography	4
Outcomes of hysteroscopy	
Surgical approach characteristics	
Hysteroscopy once	33
Hysteroscopy twice	2
Hysteroscopy once and laparotomy once	1
Complete removal of the retained products of conception	29
Menstruation recovery	
Normal	33
Decreased menstrual blood volume	3
Postoperative intrauterine adhesion	1

Thirty-three patients underwent hysteroscopy once. Two patients underwent hysteroscopy twice. The sizes of their residual products after the first hysteroscopy were 3.3 × 0.8 × 1.9 cm and 3.65 × 2.76 × 4.0 cm. These residual products were completely removed during the second hysteroscopy performed 2 weeks later. One patient with partial placenta implantation underwent hysteroscopy once and laparotomy once. The perioperative bleeding volume was 1200 mL during hysteroscopy, and the size of the residual product assessed during hysteroscopy was 4.3 × 3.2 × 2.2 cm; therefore, laparotomy was performed 1 month later. The placenta implanted into more than half of the myometrium. Of the 33 patients who underwent hysteroscopy once, the residual products of 29 patients were completely removed, but four patients had residual products. The residual product size ranged from 0.5 to 2 cm. These four patients were successfully treated with traditional Chinese medicine for 1 month to eliminate their residual products.

Clinical efficacy was also assessed according to menstruation recovery. Normal menstruation was recovered after treatment in all patients except three who experienced decreased menstrual blood volumes. Postoperative intrauterine adhesion was detected by ultrasound in only one patient who had marginal placenta previa during cesarean delivery. This patient underwent hysteroscopy twice to remove the intrauterine residual product. She experienced menstruation recovery and had no plans for pregnancy in the near future; she is still undergoing observation. Four patients had a suspected uterine arteriovenous fistula preoperatively; however, hysteroscopy confirmed three fistulas in four of the patients and RPOC in one of the patients.

## Discussion

Hysteroscopy seems to be an effective method of treating RPOC. Our study reported a complete removal rate of 80.5% (29/36) with one procedure. The normal menstruation recovery rate after 1 year of follow-up was 91.6% (33/36). A low rate of postoperative intrauterine adhesions (2.8% [1/36]) was also reported. The complications of hysteroscopic surgery can be divided into early complications, which include bleeding, uterine perforation, infection, and fluid overload, and late complications, such as incomplete resection and intrauterine adhesions [5]. A meta-analysis reported that hysteroscopy was associated with lower intrauterine adhesion rates and incomplete evacuations when compared with dilation and curettage in women with suspected RPOC [6]. Postoperative intrauterine adhesion was detected by ultrasound in one patient who had marginal placenta previa during cesarean delivery. The residual product was 5.8 × 4.9 × 5.5 cm. This patient underwent hysteroscopy twice to remove the intrauterine residual product. This patient also had decreased menstrual blood volume. Another patient who had decreased menstrual blood volume had partial placenta implantation and underwent hysteroscopy once and laparotomy once. The residual product was 6.0 × 5.9 × 5.7 cm. The last patient with decreased menstrual blood volume had a uterine arteriovenous fistula whose residual product was 2.7 × 1.8 × 2.0 cm.

**Fig. 1:**
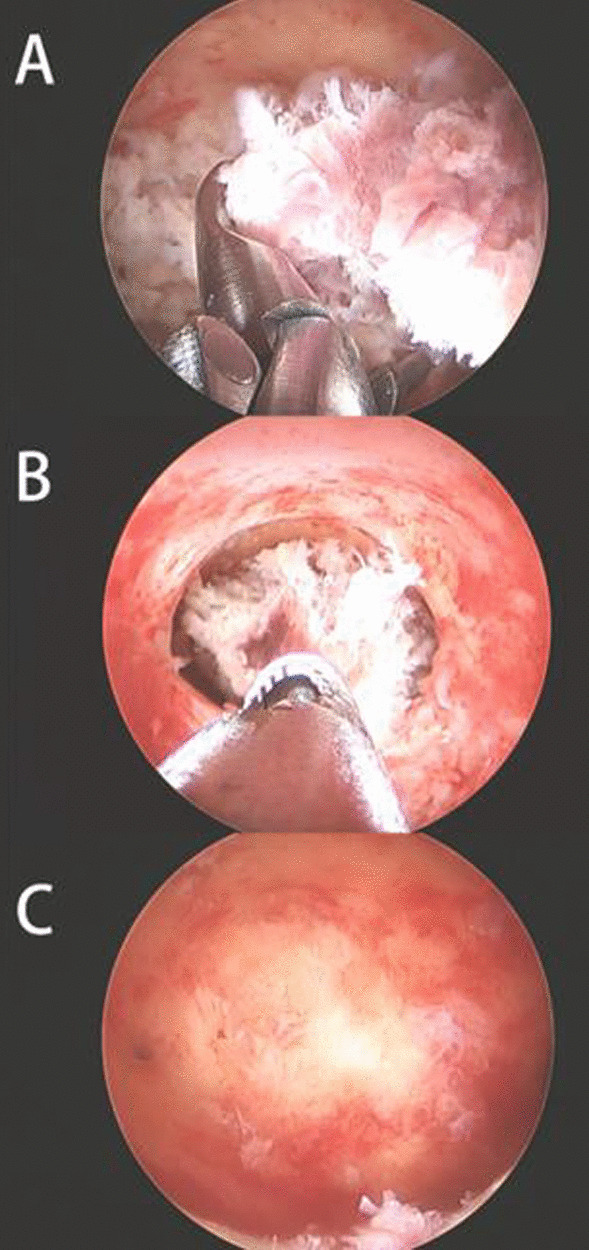
We combined cold loop resection (**a**) and hysteroscopic morcellation (**b**) in treating retained products of conception, and the retained products of conception were completely resected (**c**)

The current standard treatment method for retained products of contraception includes ultrasound-guided dilation and curettage or hysteroscopy. We chose to perform hysteroscopy for RPOC. Previous studies reported high rates of complete resection and low rates of complications for hysteroscopy performed for RPOC. Vitale et al. performed a meta-analysis of 20 studies that focused on hysteroscopy for RPOC and revealed a 91% complete resection rate, 7% incompletion rate, and 2% complication rate [7]. Rein et al. compared ultrasound-guided dilation and curettage and hysteroscopy for RPOC and reported that hysteroscopy had advantages such as fewer postoperative intrauterine adhesions and increased pregnancy rates [8]. Based on our experience, we recommend hysteroscopy for RPOC because of its high complete resection rate and low complication rate. Hysteroscopy techniques include hysteroscopic morcellation, cold loop resection, and heat loop resection. Morcellation and cold loop resection are mechanical techniques with the advantage of precise positioning, reduced damage to surrounding tissues, and the ability to reach the narrow part of the uterine horn. Studies that compared morcellation and loop excision for RPOC concluded that there were no significant differences in complete resection rates and reproductive outcomes [9, 10]. However, no clinical studies have compared the surgical outcomes of the three techniques. We believe that heat loop resection can play a role in the rapid removal of large lesions that remain in the uterine cavity. Cold knife resection and morcellation have good excision effects; therefore, they are appropriate for treating RPOC located at the uterine horn and lesions implanted in the uterine muscle layer. For complex situations, the three aforementioned methods should be combined (Fig. 1). In addition to these hysterectomy techniques, in-office hysteroscopy with the see-and-treat approach has been approved as an effective method for the management of RPOC. Nappi et al.reported that the office hysteroscope had the advantages of avoiding general anesthesia, decreasing the costs and a good compliance of patients, which also successfully used in Cesarean Scar Pregnancy combined with uterine artery embolization [11].In addition, Raz et al. compared in-office hysteroscopy with operative hysteroscopy for treating RPOC ≤ 2 cm. Operative hysteroscopy was reported to be associated with significantly more surgical complications and longer procedure and assistant times [12].

Based on our clinical experience, we recommend the time for treating RPOC. To our knowledge, no other study has shared similar experiences. RPOC associated with early-term and mid-term pregnancy should be surgically removed as early as possible to reduce the risk of bleeding and prevent intrauterine adhesions. If there is no massive vaginal bleeding after late pregnancy, hysteroscopic surgery should be delayed until the volume of the uterus shrinks after puerperium. This can reduce the surgical difficulty, operative time, bleeding, and risk of hysteroscopic water poisoning and air embolism, and improve the probability of operative success. In addition, RPOC that exist after hysteroscopic surgery are a clinical problem, and their optimal treatment method is debatable. If the residual product is small and the patient has no active vaginal bleeding, then we recommend drug treatment. However, if the remaining lesions are large and include implantation and bleeding during hysteroscopy, laparotomy can be performed to remove them.

It is important to differentiate between RPOC and uterine arteriovenous fistulas to avoid overtreatment. A uterine arteriovenous fistula comprises abnormal connections between uterine arteries and veins; it can be congenital or acquired after pregnancy or uterine surgery. Furthermore, a uterine arteriovenous fistula can cause massive bleeding. Ultrasound is the most common and convenient examination for diagnosing uterine arteriovenous fistulas. However, it cannot differentiate uterine arteriovenous fistulas from RPOC [13]. MRI and arteriography can be used to diagnose RPOC and uterine arteriovenous fistulas [14]. Based on our experience, the preoperative examination to exclude the existence of uterine arteriovenous fistula is important. Hysteroscopy can help to differentiate RPOC from uterine arteriovenous fistulas. The four patients in our study with a suspected uterine arteriovenous fistula underwent MRI, arteriography, and hysteroscopy, which verified that only three of those patients had a uterine arteriovenous fistula.

Our retrospective study elucidates the use of hysteroscopy for RPOC. However, it was limited by the small number of patients. Nevertheless, we were able to share new perspectives regarding the optimal surgical methods and time for treating RPOC as well as our experience treating residual products with hysteroscopy.

## Data Availability

All data generated or analyzed during this study are included in this article. Further enquiries can be directed to the corresponding author.

## Abbreviations

MRI: Magnetic resonance imaging
PAS: Placenta accreta spectrum
RPOC: Retained products of conception

## References

[1] Chen W, Zhang Z, Liu X (2018). Delayed surgical and non-surgical treatment of placental remnants: no difference was found in the clinical efficacy and long-term pregnancy outcomes. Ther Clin Risk Manag.

[2] Namazi G, Haber HR, Tavcar J, Clark NV (2021). Minimally invasive management of retained products of conception and the adherent placenta. Curr Opin Obstet Gynecol.

[3] Ansari SH, Bigatti G, Aghssa MM (2018). Operative hysteroscopy with the Bigatti shaver (IBS ®) for the removal of placental remnants. Facts Views Vis Obgyn.

[4] Deffieux X, Gauthier T, Menager N, Legendre G, Agostini A, Pierre F (2014). Hysteroscopy: guidelines for clinical practice from the French College of Gynaecologists and Obstetricians. Eur J Obstet Gynecol Reprod Biol.

[5] Aas-Eng MK, Langebrekke A, Hudelist G (2017). Complications in operative hysteroscopy—is prevention possible?. Acta Obstet Gynecol Scand.

[6] Hooker AB, Aydin H, Brolmann HA, Huirne JA (2016). Long-term complications and reproductive outcome after the management of retained products of conception: a systematic review. Fertil Steril.

[7] Vitale SG, Parry JP, Carugno J, Cholkeri-Singh A, Della Corte L, Cianci S (2021). Surgical and reproductive outcomes after hysteroscopic removal of retained products of conception: a systematic review and meta-analysis. J Minim Invasive Gynecol.

[8] Rein DT, Schmidt T, Hess AP, Volkmer A, Schöndorf T, Breidenbach M (2011). Hysteroscopic management of residual trophoblastic tissue is superior to ultrasound-guided curettage. J Minim Invasive Gynecol.

[9] Van Wessel S, Coryn N, van Vliet H, Schoot B, Weyers S, Hamerlynck T (2020). Reproductive and obstetric outcomes after hysteroscopic removal of retained products of conception. J Minim Invasive Gynecol.

[10] Hamerlynck TW, van Vliet HA, Beerens AS, Weyers S, Schoot BC (2016). Hysteroscopic morcellation versus loop resection for removal of placental remnants: a randomized trial. J Minim Invasive Gynecol.

[11] Sorrentino F, De Feo V, Stabile G, Tinelli R, D'Alterio MN, Nappi L (2021). Cesarean scar pregnancy treated by artery embolization combined with diode laser: a novel approach for a rare disease. Medicina (Kaunas).

[12] Raz N, Sigal E, Gonzalez Arjona F, Calidona C, Garzon S, Uccella S (2022). See-and-treat in-office hysteroscopy versus operative hysteroscopy for the treatment of retained products of conception: a retrospective study. J Obstet Gynaecol Res.

[13] Guan D, Wang J, Zong L, Li S, Zhang YZ (2017). Acquired uterine arteriovenous fistula due to a previous cornual pregnancy with placenta accreta: a case report. Exp Ther Med.

[14] Hong W, Wang BY, Wu ZP, Gao F, Li SD, Li XC (2020). Systematic retrospective analysis of 13 cases of uterine arteriovenous fistula: pathogeny, diagnosis, treatment and follow-up. J Obstet Gynaecol Res.

